# Completion of Upper Secondary Mainstream School in Autistic Students in Sweden

**DOI:** 10.1007/s10803-024-06470-8

**Published:** 2024-07-17

**Authors:** Isidora Stark, Jessica E. Rast, Michael Lundberg, Nora Döring, Anna Ohlis, Selma Idring Nordström, Dheeraj Rai, Cecilia Magnusson

**Affiliations:** 1 Department of Neurobiology, Care Sciences and Society, Karolinska Institutet, Stockholm, Sweden; 2 A.J. Drexel Autism Institute, Drexel University, Philadelphia, PA USA; 3 Department of Global Public Health, Karolinska Institutet, Stockholm, Sweden; 4 Centre for Epidemiology and Community Medicine, Region Stockholm, Stockholm, Sweden; 5 Department of Clinical Neuroscience, Karolinska Institutet, Stockholm, Sweden; 6 Centre for Academic Mental Health, Population Health Sciences, Bristol Medical School, University of Bristol, Bristol, UK; 7 NIHR Biomedical Research Centre, University of Bristol, Bristol, UK; 8 Bristol Autism Spectrum Service, Avon and Wiltshire Partnership NHS Mental Health Trust, Bristol, UK

**Keywords:** Autism, Inclusive education, ADHD, Educational outcomes, Total-population study

## Abstract

Higher education is an increasingly necessary achievement to attain employment. However, even in cases where a student has the academic skills to succeed, educational environments may not support students across all other domains necessary for education success, including social and communication needs. This is especially true for students with disabilities and autistic students, where the rate of completion of non-compulsory education is unknown. We used the Stockholm Youth Cohort (children aged 0–17 years from 2001 to 2011), a total population cohort (*N* = 736,180) including 3,918 autistic individuals, to investigate the association between autism without intellectual disability and completion of upper secondary education. We assessed the impact of sex and co-occurring Attention-Deficit/Hyperactivity Disorder (ADHD) on this association. By age 20 years (the expected age of completion), 68% of autistic students and 91% of non-autistic students admitted to upper secondary education had completed. In logistic regression models adjusted for student demographics, autistic students had almost five-fold higher odds of not completing secondary school (OR 4.90, 95% CI 4.56 5.26) compared to their non-autistic peers. Autistic students with ADHD had particularly high odds of non-completion of upper secondary school. Autistic students without intellectual disability attending mainstream education are substantially less likely to complete upper secondary education as compared to their peers. These findings have implications for the appraisal of how inclusive school policies serve autistic students’ academic and social needs, ultimately addressing population health and independent living.

## Introduction

Higher education is increasingly required to be competitive for jobs and to maintain economic stability and productive employment (Trostel, 2015). Many people with disabilities have the academic capability to be successful in education, but they may require academic and non-academic supports to be successful in school. Autism is a developmental condition characterized by difficulties in reciprocal social communication and social interaction, restricted interests, and repetitive behaviors (American Psychiatric Association, 2013). Autistic students may require a variety of educational accommodations or supports to be successful; support in social, communication, time management, and organization are integral to autistic student success (Anderson et al., 2018, 2019). Autism is a common neurodevelopmental condition and the world-wide prevalence is estimated to be 1-1.5% (Lyall et al., 2017; Myers et al., 2019).

Education systems vary by country, but higher education generally encompasses non-compulsory education in older children and young adults. This often includes postsecondary or tertiary education such as college or university. In Sweden, higher education also includes upper secondary education, which typically occurs between the ages of 15–20 (after the 9th year of schooling), is non-compulsory, but is an entitlement. Successful completion of upper secondary education is considered a necessary requisite for further education or skilled manual work.

Since 2009, almost all autistic students without intellectual disability (ID) are included in mainstream education in Sweden in primary, secondary, and upper secondary education (Berhanu, 2011). Some students may go to resource schools that provide adapted education, but these schools follow mainstream school curriculum. Students with ID are instead offered to follow a separate curriculum. The Swedish Education Act promotes adaptation and special support to all students who risk not reaching knowledge requirements in education, but the law does not mandate or specify the support actions (Göransson et al., 2011).

While the rates of upper secondary educational completion among autistic students is unknown, a small evidence base examines related education indicators that inform the importance of examining this question across contexts. A recent Swedish study examined final grades at the 9th year of schooling, the end of compulsory education and crucial for qualification to upper secondary education, and found that autistic students were considerably less likely to qualify compared to their non-autistic peers (57% vs. 86%, respectively) (Stark et al., 2021). In contrast, a Danish cross-sectional study reported that autistic and non-autistic students did not differ in educational outcomes at the end of compulsory education (Toft et al., 2021). In the early 2000’s, 39% of autistic students enrolled in special education in secondary school in the United States attended some sort of postsecondary education, as did 30% of a sample of autistic people born between 1974 and 1984 in British Columbia, Canada (Eaves & Ho, 2008; Roux et al., 2015). A cross-sectional study investigating educational outcomes in tertiary education in 185 autistic students in Germany found that the proportion of young adults having university qualification was higher among those with autism diagnosed in adulthood (56.8%) compared to the general population (29.5%) (Frank et al., 2018). However, the authors acknowledge that their sample likely captured autistic adults with good adaptive and social skills (Frank et al., 2018). Graduation rates from postsecondary or higher education are generally unknown. While educational systems vary across countries, the investigation of academic success in autistic students is an important measure of universal need for educational support in this population.

Considering the robust association between educational completion and socioeconomic advantages later in life, it is important to understand the support needs of autistic students to maximize their success in higher education (Braveman & Gottlieb, 2014; Eide & Showalter, 2011; Gustafsson et al., 2010). This is also an issue of growing importance, as a larger group of autistic students attend mainstream schooling now than ever before (Berhanu, 2011; Dillenburger et al., 2015).

There are other considerations for autistic students that may alter chances of educational completion, including co-occurring diagnoses and demographic characteristics. Attention-Deficit/Hyperactivity Disorder (ADHD) and autism frequently co-occur and both conditions may impact educational completion (Antshel & Russo, 2019; Ronald et al., 2014). However, it is unclear how they interact and the role of ADHD in educational outcomes of autistic students is poorly understood. A study from Taiwan indicates worse academic performance in autistic students with co-occurring ADHD as compared to those without ADHD (Chiang et al., 2018). There is limited research on the impact of sex on educational completion in autism. In studies in general populations, females largely have better educational outcomes than males, particularly among groups with disadvantaged socio-economic backgrounds (Delaney & Devereux, 2021; Fortin et al., 2015; Serbin et al., 1990, 2013). This female advantage manifests from early schooling through higher education (Fortin et al., 2015; Serbin et al., 2013). In contrast to findings in the general population, a recent study on elementary education in Sweden found autistic girls underperformed in relation not only to neurotypical girls and boys, but also in relation to autistic boys (Stark et al., 2021). However, other studies suggest autistic girls may have advantages in comparison with their male peers, such as superior executive functioning and less stereotypic behaviors, that may promote school performance (Bölte et al., 2011).

The current population-based study aims to investigate the association between autism without ID and educational completion in upper secondary school using prospectively collected data from Swedish national and regional registers. In addition, we investigate the roles of co-occurring ADHD and sex in upper secondary educational completion in autistic students.

## Methods

### Study Population and Design

**Fig. 1:**
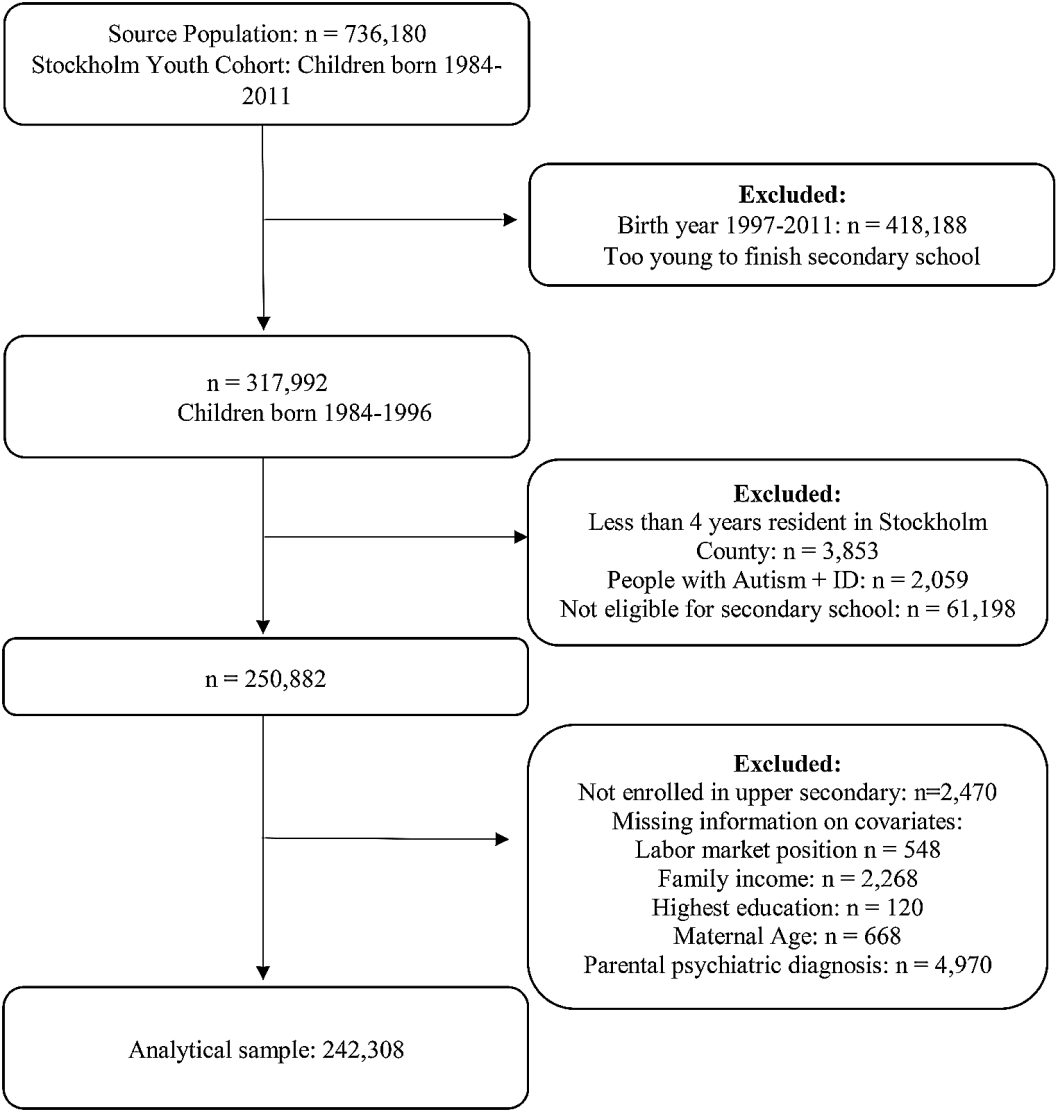
Derivation of study population

This study used linked national and regional Swedish population registers to create a longitudinal total population cohort of children aged 0–17 years residing in Stockholm County, Sweden, from 2001 to 2011 (*N* = 736,180) (the Stockholm Youth Cohort (SYC) (Ludvigsson et al., 2009). SYC includes prospectively compiled data on individuals and their first-degree relatives (Idring et al., 2012). This study restricted SYC to individuals born before 1997 and therefore old enough to complete upper secondary school (*n* = 418,188). Individuals who resided less than 4 years in Stockholm County were excluded to allow a reasonable opportunity for health care services to establish any diagnosis of autism. Autistic individuals with ID were excluded from this study as very few attend mainstream education in Sweden. Contemporary estimates suggest that around 25% of individuals with autism also present with intellectual disability (ID) (Idring et al., 2012). We excluded those we defined as not eligible for upper secondary education if they failed to pass all three core subjects (mathematics, Swedish, and English) or if they had no registered grades in the year of final exams (*n* = 61,198). After excluding those who did not enroll in upper secondary school (*n* = 2,470) and those with missing covariate information (*n* = 8,574), our final analytical sample consisted of 242,308 individuals eligible as depicted in Fig. 1.

### Independent Variables

Autism diagnoses were identified from registers covering all services providing diagnostic evaluations and care for autism in Stockholm, using International Classification of Diseases (ICD) and Diagnostic and Statistical Manual of Mental Disorders (DSM-IV or DSM-V) codes (ICD-9-299, ICD-10 F84) and by the denotation of autism by habitation services (Axén et al., 2010). DSM-IV and DSM-V criteria were used based on the year of diagnosis, reflecting the age of the cohort (born between 1984 and 1997). This multisource methodology to ascertain cases with autism has high validity (Idring et al., 2012). Diagnoses of ID and ADHD were identified in the same manner (defined as ICD-9-317–319 and ICD-10-F70–F79 for ID; and ICD-9-314.00-314.01 and ICD-10-F90.0, F98.8 for ADHD).

Sex was also a variable of interest in this study, as it has been shown to be predictive of educational success in general student populations (Serbin et al., 2013). Swedish registers do not provide information on gender, thus only data regarding sex assigned at birth (binary variable female/male) was used in this study.

### Outcome

The outcome of interest in this study was completion of upper secondary education (binary variable), ascertained by having a final exam grade registered before or during the year the individual turned 20. Age 20 as the upper age cut off was based on the mean age for completion of this educational level registered by Statistic Sweden, and very few complete after this age. Information was retrieved by the Swedish Longitudinal Integration Database for Health Insurance and Labour Market Studies (LISA) that annually integrates data on education, labor market and social sectors for all individuals living in Sweden (since 1990); and the National School Register (UREG), which holds information on individual school performance collected from all municipal and independent schools since 1988 (Ludvigsson et al., 2019).

### Covariates

We included variables shown in previous literature to be associated with autism and educational completion, including variables that capture parental socioeconomic position such as migration status, family income, and parental education (Chiang et al., 2012; Galobardes et al., 2007). Sociodemographic characteristics were collected from various registers as previously described (Idring et al., 2012). In the analyses we included parents’ ages at child’s birth (Idring et al., 2015), immigration status (born in Sweden with at least one Swedish-born parent, born in Sweden with both parents born abroad, and born outside Sweden) (Bankston III & Zhou, 2002), familial income (grouped into quintiles after deducting taxes and adjusting for family size and inflation, with 1 being the lowest quintile and 5 the highest), and parents’ educational level at child’s birth (Sirin, 2005). The highest educational level for either parent was grouped as primary (9 years of schooling or less), upper secondary (10–12 years), and higher (> 12 years). Given the evidence that parental psychiatric conditions may influence both autism (Daniels et al., 2008) and school performance in their children (Ranning et al., 2018), history of any maternal and paternal psychiatric care was retrieved and analyzed as a dichotomous variable. We excluded individuals with missing data on these covariables and did not conduct missing data imputation (about 3% of observations). Missing data on these variables was more common in autistic students with parents born outside of Sweden.

### Statistical Analysis

First, we calculated proportions for (1) all students, (2) autistic students, and (3) students without autism over all covariates. Second, we used logistic regression to assess the association between autism and completion of upper secondary education separately by sex. The first model adjusted for year of birth within the cohort. The second model additionally adjusted for parental covariates of maternal and paternal age, family income, highest parental education level, migration status, and parental psychiatric diagnosis. The third model additionally adjusted for ADHD status. All models were run separately by sex. The final analysis separately examined all previous models by ADHD status by repeating the series logistic regression models stratified both by sex and ADHD status. Analyses were performed using SAS version 9.4.

## Results

Sociodemographic characteristics of the study population and non-completion of upper secondary education in mainstream schools are presented in Table 1. Autistic students (*n* = 3,918) were more likely to be male (62.0%), born after 1992 in Sweden to parents born in Sweden, and have parents who had a history of psychiatric diagnosis compared to non-autistic students. In our cohort, 44.4% of students with autism also had a diagnosis of ADHD, compared to 4.3% of students without autism.

**Table 1:** Descriptive characteristics of the study population

	Total	Autism	No autism
	(*N*=242,308)	(*N*=3,918)	(*N*=238,390)
	% / mean (SD^a^)	% / mean (SD^a^)	% / mean (SD^a^)
**Male**	50.2	62.0	50.0
**Birth year**			
1984-1987	25.8	18.0	25.9
1988-1991	32.6	30.3	32.7
1992-1996	41.6	51.7	41.4
**Maternal age at birth**	29.1 (5.1)	29.2 (5.3)	29.1 (5.1)
**Country of Birth**			
Outside of Sweden	4.4	1.8	4.4
Sweden with foreign born parents	13.1	8.6	13.1
Sweden with at least one Swedish born parent	82.6	89.6	82.4
**Highest parental education**			
9 years or less	6.6	4.8	6.7
10-12years	43.2	44.3	43.2
13 years or more	50.2	51.0	50.1
**Family income at birth (quintiles)**			
1^st^ (lowest)	17.9	14.5	17.9
2^nd^	18.9	20.0	18.9
3^rd^	20.2	22.8	20.1
4^th^	21.1	21.5	21.1
5^th^ (highest)	22.0	21.3	22.0
**Any maternal psychiatric diagnosis**	43.6	59.4	43.4
**Any paternal psychiatric diagnosis**	28.0	36.9	27.9
**Non-completion of upper secondary education**	9.5	32.3	9.1

^a^SD=Standard deviation

### Completion of Upper Secondary Mainstream Education

By the year the student turned 20, 9.5% of non-autistic students had not completed upper secondary mainstream education, compared to 32.3% of autistic students who did not achieve this educational goal. The associations between autism and non-completion of upper secondary mainstream education are displayed stratified by sex in Table 2. Crude analyses (Model 1, adjusted for year of birth) showed that autistic students had substantially higher odds of non-completion compared to their non-autistic peers (OR 5.02, 95% *CI* 4.70, 5.38). Autistic males had more than four-fold odds of not completing upper secondary mainstream education compared to non-autistic ones (OR 4.30, 95% CI 3.93, 4.70). Among females, the odds ratio was larger (OR 6.46, 95% CI 5.78, 7.21). The effect size attenuated slightly after adjustments for socioeconomic familial factors (Model 2) for males and females, respectively, 4.24 (95% CI 3.87, 4.65) and 6.19 (95% CI 5.53, 6.93). In the third model, adjustment for ADHD further attenuated effect size in both male and female students, yet the odds of non-completion remained elevated in autistic students (Model 3) (female OR = 3.46, 95% CI 3.06, 3.91 and male OR = 2.63, 95% CI 2.39, 2.91). To further test whether sex modified the effect of autism on the outcome in all analyses, we included an interaction term of sex and autism status in a non-sex-stratified logistic model. A significant modification of the effect of autism by sex was observed as a statistically significant interaction term in all models (p-value < 0.0001).

**Table 2:** Association between autism without intellectual disability (ID) and non-completion of upper secondary education in mainstream schools, overall and by sex

	Non-completers / Completers (*N*)	Model 1 OR (95% CI)	Model 2 OR (95% CI)	Model 3 OR (95% CI)
**All individuals**				
No autism	21,693 / 238,390	*Ref.*	*Ref.*	*Ref.*
Autism	1,267 / 3,918	5.02 (4.70, 5.38)	4.90 (4.56, 5.26)	2.91(2.70, 3.15)
**Males**				
No autism	12,168 / 119,128	*Ref.*	*Ref.*	*Ref.*
Autism	758 / 2,427	4.30 (3.93, 4.70)	4.24 (3.87, 4.65)	2.63 (2.39, 2.91)
**Females**				
No autism	9,525 / 119,262	*Ref.*	*Ref.*	*Ref.*
Autism	509 / 1,491	6.46 (5.78, 7.21)	6.19 (5.53, 6.93)	3.46 (3.06, 3.91)

Model 1 adjusted for year of birth; Model 2 additionally adjusted for maternal age at birth, family income, highest parental education, migration status, and maternal and paternal psychiatric diagnosis; Model 3 additionally adjusted for ADHD diagnosis

### Autism, ADHD, and Non-Completion of Upper Secondary Mainstream Education

Results on the association between autism and non-completion of mainstream upper secondary schooling based on co-occurring ADHD status are shown in Table 3. Overall, we found high odds of non-completion in all groups but observed a gradient in ORs for non-completion, highest in the group with autism and ADHD (OR = 6.03,95% CI 5.44, 6.69), followed by the group with only autism (OR = 4.99, 95% CI 4.53–5.50), then the group with only ADHD (OR = 3.95, 95% CI 3.74, 4.15) compared to youth without these conditions. Associations were most pronounced in autistic females with both diagnoses (OR = 7.07, 95% CI 6.00, 8.31).

**Table 3:** Association between autism without intellectual disability (ID), attention deficit hyperactivity disorder (ADHD) and non-completion of upper secondary mainstream education, overall and by sex

	Non-completers / Completers (*N*)	Model 1	Model 2
		OR (95% CI)	OR (95% CI)
**All individuals**			
No autism/no ADHD ADHD only Autism only Autism/ADHD	19,320 / 229,758 2,373 / 8,632 652 / 2,178 615 / 1,740	*Ref.* 4.51 (4.29, 4.75) 4.97 (4.53, 5.46) 6.37 (5.78, 7.04)	*Ref.* 3.95 (3.74, 4.15) 4.99 (4.53, 5.50) 6.03 (5.44, 6.69)
**Males**			
No autism/no ADHD	10,851 / 114,493	*Ref.*	*Ref.*
ADHD only	1,317 / 4,635	4.19 (3.91, 4.48)	3.71 (3.46, 3.97)
Autism only	392 / 1,377	4.16 (3.69, 4.69)	4.19 (3.70, 4.73)
Autism/ADHD	366 / 1,050	5.61 (4.92, 6.39)	5.44 (4.76, 6.22)
**Females**			
No autism/no ADHD	8,469 / 115,265	*Ref.*	*Ref.*
ADHD only	1,056 / 3,997	4.97 (4.61, 5.36)	4.26 (3.95, 4.60)
Autism only	260 / 801	6.73 (5.79, 7.83)	6.75 (5.78, 7.87)
Autism/ADHD	249 / 690	7.71 (6.58, 9.04)	7.07 (6.00, 8.31)

Model 1 adjusted for year of birth; Model 2 additionally adjusted for maternal age at birth, family income, highest parental education, migration status, and maternal and paternal psychiatric diagnosis

## Discussion

To our knowledge, this is the first longitudinal total-population study investigating completion of upper secondary mainstream education in autistic students without ID. We demonstrated that autistic students in Stockholm County had an almost five-fold higher odds of non-completion of upper secondary education by age 20, compared to their non-autistic peers. The strong association between autism and noncompletion of upper secondary education was not explained solely by co-occurring ADHD, in fact, autistic students without ADHD had higher odds of non-completion than non-autistic students with ADHD.

The results of the current study are consistent with previously described difficulties in autistic students (Shattuck et al., 2012; Toft et al., 2021), and they expand more general findings on educational underperformance in this group (Keen et al., 2016; Levy & Perry, 2011). Studies from the 20th century estimated that approximately 50–60% of autistic students leave school without formal academic or vocational qualifications (Levy & Perry, 2011). Our investigation in more recent birth cohorts suggests that the inequalities in educational completion in autistic students, and thus chances for later employment and social and economic independence, seem to persist despite increased awareness and policies on educational access for all students.

Our results further suggest that there is no female advantage of educational achievement in autistic students, in contrast to findings in the general population where females show a relative advantage in educational achievements compared to males (Serbin et al., 2013). We found that the odds of non-completion were higher for autistic females compared with non-autistic females than for autistic males compared to non-autistic males. Autistic females are often diagnosed at an older age (Idring et al., 2012), which might partially explain negative outcomes as timely support may be missed. They may also be likely to have other mental health comorbidities further disadvantaging their educational attainment. Previous research on sex differences in autism reported that diagnosed females have generally greater impairment in social and communication skills and cognitive ability (Frazier et al., 2014), which may partly explain the observed underperformance relative to non-autistic females. This raises the question whether an autistic female phenotype with females with less social, communication, and intellectual impairment is differentially under identified. Social camouflaging (the use of strategies to compensate for and mask autistic characteristics in social situations) has been found to be present to a greater degree in autistic females than males, which may lead to underdiagnosis in females camouflaging (Hull et al., 2017, 2020).

While autistic students may have many academic strengths, including technical skills, memory, and attention to detail, academic performance could be hindered by difficulties in non-academic skills that are integral to academic success (Anderson et al., 2018, 2019). These include social interactions, verbal and non-verbal communication, and organization and time management (Jansen et al., 2017). For many students with autism, attendance at a mainstream school may be appropriate, but not without educational and social support either via an inclusive school or individualized supports. While Swedish education policies aim to be inclusive, the evolution of Swedish inclusive school policy from a focus on inclusive school environments to a focus on individual student metrics of success may be impacting student achievement. The balance between creating an accessible mainstream environment and the focus on individualized support is a delicate one that Swedish policy has inconsistently dictated over the past several decades (Isaksson & Lindqvist, 2015), even as one of the first countries to adopt the policy of the right of adequate education for all individuals (Berhanu, 2011). What is evident from this study is that the current support system is not working for autistic students as measured by success in timely educational attainment.

## Strengths and Limitations

There are several notable strengths to this study. The total-population design with data prospectively collected from records kept by government agencies or health and official institutions ensures minimal risk of selection, recall, and report biases. This approach of case-finding has been validated in our previous research (Idring et al., 2012). The study included a large contemporary sample where autism was comprehensively ascertained. The study benefited from a long-term follow-up period and minimized confounding by having rich data on familial variables. Finally, this is the first large study with a long follow-up to assess objectively measured indicators of educational outcomes.

The results also need to be interpreted within several limitations. First, register data used in the study do not include information on student-specific factors such as intelligence, behavioral difficulties, or the supports or adaptations available, all of which could have an impact on educational completion. Although IQ can predict educational outcomes, some evidence also indicates that academic skills in autistic students may be higher than expected by their intelligence, which may not make IQ a reliable predictor of educational success (Estes et al., 2011; Kim et al., 2018). While we excluded students with intellectual disability (ID) from this study, students with unidentified ID may inadvertently have been included if they followed a mainstream curriculum and attained qualifying exam grades (study inclusion criteria). In this case, their inclusion may not much impact study findings, as their academic performance is adequate for upper secondary attendance. Second, we could not investigate school related factors that may impact school performance, such as size and type of classes, attitudes, peer-relations in the classroom, or teachers’ competence and skills for working with autistic students. If students differentially attend varying school types, this could influence results. Third, data on treatment for ADHD, which has been showed to improve educational results in previous research and is generally common in autistic children and adolescents with ADHD, were not available for this study (Boland et al., 2020; Rast et al., 2021).

## Conclusion

Results from this contemporary total population study indicate that students with autism without ID, especially those with co-occurring ADHD and females, are significantly less likely to successfully complete upper secondary education than their non-autistic peers by age 20. Thus, educational disadvantages, and the resulting diminished chances of social and economic independence, remain among autistic individuals today despite the widened awareness and increased diagnosis of autism. These findings have implications for the appraisal of how supports are provided to autistic students in upper secondary education, ensuring they have the opportunity to gain the education required to enter the competitive labor market and obtain the desired level of employment. Exploration of the best educational support systems may be informed by research into students’ own experience of upper secondary schooling.
